# Infection-Induced Systemic Inflammation Is a Potential Driver of Alzheimer's Disease Progression

**DOI:** 10.3389/fnagi.2019.00122

**Published:** 2019-05-28

**Authors:** Vijayasree V. Giridharan, Faisal Masud, Fabricia Petronilho, Felipe Dal-Pizzol, Tatiana Barichello

**Affiliations:** ^1^Department of Psychiatry and Behavioral Sciences, Translational Psychiatry Program, McGovern Medical School, The University of Texas Health Science Center at Houston, Houston, TX, United States; ^2^Department of Anesthesiology, Houston Methodist Hospital, Houston, TX, United States; ^3^Health Sciences Unit, Laboratory of Neurobiology of Inflammatory and Metabolic Processes, Graduate Program in Health Sciences, University of South Santa Catarina, Tubarão, Brazil; ^4^Laboratory of Experimental Pathophysiology, Graduate Program in Health Sciences, University of Southern Santa Catarina, Criciúma, Brazil; ^5^Graduate Program in Health Sciences, Laboratory of Neurosciences, Health Sciences Unit, University of Southern Santa Catarina (UNESC), Criciúma, Brazil

**Keywords:** infection, systemic inflammation, neuroinflammation, Alzheimer's disease, cognition

The cases that are categorized as familial Alzheimer's disease (AD) account for 5% of the total AD cases, whereas sporadic cases account for 95% (Masters et al., 2015; Baker et al., 2018). Among the different risk factors underlying sporadic cases of AD, infection might play a role in late-onset AD. Over the past three decades, infectious agents such as bacteria, viruses, fungi, and protozoa have been reported to trigger the development of AD (Sochocka et al., 2017). The infection hypothesis is not a recent idea; the involvement of microorganisms in AD progression was proposed by Aloysius Alzheimer (Fulop et al., 2018). In the 1990s, three laboratories from different countries associated the infection with the etiology of AD. Elderly patients infected with herpes simplex virus (HSV)-1 developed toxic accumulation of amyloid β (Aβ) and phosphorylated (p)-tau protein in the brain (Itzhaki et al., 2016). In autopsy cases with histopathologically confirmed AD, spirochetes were found in blood, cerebrospinal fluid, and brain tissue (Miklossy, 1993). A third study by Balin et al. reports that *Chlamydia pneumoniae* was present in post-mortem brain samples from patients with AD (Balin et al., 1998). In another study, systemic infection by *C. pneumoniae*, a Gram-negative bacterium, was associated with a 5-fold increase in AD occurrence, and in many AD patients, elevated anti-*C. pneumoniae* titers in blood have also been reported (Balin et al., 2008). The result from an association study of 128 AD patients and 135 healthy controls provides evidence of infectious burden, comprising viruses, and bacteria, that is associated with AD (odds ratio ~4) (Bu et al., 2015). A national representative survey of US residents involving 1,194 patients with 1,520 hospitalizations for infection with severe sepsis revealed that sepsis survivors were independently associated with substantial and persistent new cognitive impairment and functional disability (Iwashyna et al., 2010). All of these studies support the notion that infectious etiology might be a causative factor for the inflammatory pathway associated with AD progression.

The accumulation of misfolded Aβ in the brain has been proposed to be the critical triggering event in a complex pathophysiological cascade that leads to AD pathology. The additional physiological role of Aβ as an antimicrobial agent in *in vitro* and *in vivo* models has been shown by Robert Moir and Rudolph Tanzi (Soscia et al., 2010). In both rodent and nematode models, the authors reported the antimicrobial properties of the Aβ peptide. Transgenic mice expressing the human mutant form of APP were infected with *Salmonella enterica*; the nematode *Caenorhabditis elegans* expressing the human Aβ_42_ peptide were infected with *Candida albicans*. The mice and *C. elegans* expressing the Aβ peptide survived longer than did the control group without Aβ expression after infection. In another Aβ-overexpressing mouse model, *S. typhimurium* injection in the brain resulted in the induction of Aβ amyloid deposits with an extended survival rate. These studies also suggested that Aβ oligomerization, which is considered a pathological development in the context of neurodegeneration, may be a necessary step to potentiate the antimicrobial activity of the peptide (Kumar et al., 2016). These results raised some important questions about the association between AD and microbial infection. The authors also unveiled the mechanism by which Aβ elicits its antimicrobial property. Aβ binds to a microbe and entraps it by forming amyloid fibrils. The presence of microbes serves as an efficient surface for nucleation of amyloid aggregates, thereby raising the possibility of amyloid deposition (Golde, 2016) (Figure 1). Thus, brain infection in a mouse model of AD triggered formation of Aβ plaques earlier than they usually developed. The above reports on neuroinflammation-mediated neurodegeneration and the role of Aβ as an antimicrobial agent have impelled the emanation of the “antimicrobial protection hypothesis” (Moir et al., 2018) in addition to different hypotheses concerning development of AD, including the cholinergic hypothesis, amyloid hypothesis, tau hypothesis and inflammatory hypothesis (Du et al., 2018). Even so, the findings raise the question of how the protective function of Aβ fails. The possible answer is microglial dysfunction; accumulation of biologically active peptides following an infection might have not been effectively cleared by microglia in the brain of patients with AD (Stilling and Cryan, 2016) (Figure 1). Additionally, Aβ accumulation in the brain may act as an early toxic event in the pathogenesis of AD. The Aβ monomers, soluble and probably nontoxic, would aggregate into different complex assemblies, including soluble oligomers and protofibrils, with various degrees of toxicity. That may spread throughout the brain, and eventually developed into insoluble amyloid fibrils further assembled into amyloid plaques, which are one of the characteristic histological lesions on AD brains. In the context of AD, the biological significance of Aβ conformational states is important as the different types of assemblies might differentially influence the development of neurodegenerative stages (Miklossy, 2011; Tycko, 2015; Chen et al., 2017). Hence, it would be extremely important to gain knowledge on Aβ conformational changes following infection that potentially affect the central nervous system (CNS).

**Figure 1:**
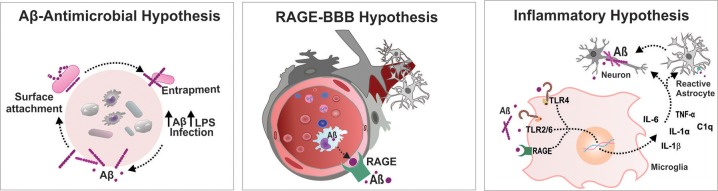
Infectious etiology triggers AD progression. Aβ-Antimicrobial hypothesis: Aβ binds to a microbe and entraps it by forming amyloid fibrils, thereby raising the possibility of amyloid deposition. RAGE-BBB hypothesis: RAGE acts as an important transporter via regulating influx of circulating Aβ into brain. Inflammatory hypothesis: Systemic inflammation increases the BBB permeability and activates microglia cells triggering Aβ deposition in the brain. Aβ, amyloid beta; BBB, blood brain barrier; IL, interleukin; LPS, lipopolysaccharide; RAGE, receptor for advanced glycation end products; TLR, toll-like receptor; TNF, tumor necrosis factor.

Recently, the results from three different groups of investigators demonstrated that sepsis, a life-threatening acute organ dysfunction due to a dysregulated host immune response after infection, induces systemic inflammation that exacerbates the accumulation of Aβ and triggers AD progression. A study by Gasparotto et al. reported that sepsis induction in a cecal ligation and perforation model escalated the levels of Aβ, p-tau protein and receptor for advanced glycation end products (RAGE) markers with simultaneous cognitive impairment in wild-type rats. The increase in AD markers was accompanied by activation of microglia and astrocytes (Gasparotto et al., 2018). Another study by Wang et al. demonstrated that the induction of sepsis in a lipopolysaccharide (LPS) endotoxemia model upregulated the levels of soluble monomeric Aβ (1–42) and p-tau. The levels of the inflammatory markers, interleukin (IL)-1β, IL-6, and tumor necrosis factor-α (TNF-α) and cortical microglial density, increased after systemic injection of LPS (Wang et al., 2018). The third study by Ehler et al. demonstrated staining of β-amyloid precursor protein (APP) in the post septic rat brain after experimental sepsis induction by fecal peritonitis, and demonstrated staining for β-APP in the postmortem septic brain (Ehler et al., 2017). Together, all of these reports suggest that inflammation is a cardinal component of the pathophysiology of sepsis. Thus, the role of inflammation might be associated with the long-term cognitive impairment observed in sepsis survivors.

A compromised blood-brain barrier (BBB) is one of the consequences after bacterial and viral infections, which leads to diffuse cerebral dysfunction after the systemic inflammatory response, with or without direct CNS infection (Cain et al., 2017; Al-Obaidi and Desa, 2018). Increased BBB permeability drives significant alteration in consciousness, facilitating the storm of pro-inflammatory cytokines in the CNS that leads to brain dysfunction. Infection-induced systemic inflammation provokes microbiome dysbiosis in response to pathogenic microorganisms and/or as a result of altered immune function. Altered immune function after infection acutely exacerbates the peripheral load of cytokines. The systemic inflammation-induced BBB breach escalates the transportation of a number of pro- and anti-inflammatory cytokines and chemokines to the brain, including TNF-α, IL-1β, transforming growth factor beta (TGF-β), and monocyte chemoattractant protein 1 (MCP1) (Semmler et al., 2008). An increased level of the systemic inflammatory marker TNF-α was demonstrated to be associated with an increase in cognitive decline in AD patients (Holmes et al., 2009). Recent reports demonstrate that in a *Drosophila* model, *Enterobacteriaceae* family infection exacerbates the progression of AD by promoting immune hemocyte migration to the brain (Wu et al., 2017). Additionally, polymicrobial infection-induced RAGE accumulation facilitates the transport of the Aβ peptide across the BBB and increases the central Aβ load (Gasparotto et al., 2018) (Figure 1). Therefore, endothelial activation followed by BBB alteration modulates the transport of potential neurotoxic components from the peripheral circulation to the cerebral compartment, which facilitates the neuroinflammatory cascade of AD.

Recent evidence from both preclinical and clinical studies suggests the activation of microglia after CNS infection by viruses, bacteria, fungi and parasites (Rock et al., 2004; Ashraf et al., 2018). Microglia, an indicator of brain inflammation, have multiple facets for neuroinflammation, including cytotoxicity, repair, regeneration, and immunosuppression, due to their ability to acquire diverse activation states or phenotypes (Chhor et al., 2013). During infection, microglia express immunoreceptors (IRs), which are capable of recognizing foreign molecules and triggering innate immune responses. Pattern-recognition receptors (PRRs), one of the examples of IRs, are the central components of the innate immune system that recognize danger signals, such as invading bacteria, and initiate the immune response. PRRs recognize conserved pathogen molecular structures, commonly known as pathogen-associated molecular patterns (PAMPs), and intracellular molecules released from damaged host cells, collectively known as damage-associated molecular patterns (DAMPs) (Linnartz and Neumann, 2013). The PRRs that trigger amyloidosis include TLRs, RAGE, cluster of differentiation (CD)14, and purine receptors (P2X7). The biologically active Aβ binds to these receptors and upregulates the Aβ load in the CNS. A recent systematic review and meta-analysis concluded that inhibition of RAGE, a danger signal that triggers the inflammatory response, improves outcomes after systemic inflammation in animal models (Zhao et al., 2018). Intriguingly, the study by Keren-Shaul et al. identified an unexpected population of microglia called disease-associated microglia (DAM) using single-cell RNA sequencing technology and demonstrated its significance relevant to AD pathology (Keren-Shaul et al., 2017). A recent report revealed that pro-inflammatory microglia secrete IL-1α, TNF, and C1q, and these cytokines are sufficient to activate astrocytes termed A1 reactive astrocytes. The A1-reactive astrocytes produce complement components that release toxic factors that, in turn, damage neurons, and oligodendrocytes, thereby contributing to the cognitive decline (Clarke et al., 2018). To understand how infection induces brain dysfunction, deep insights into brain-immune cross talk are required, which can be achieved by identifying the role of DAM and reactive astrocytes after infection. Together, all these findings support the “inflammation hypothesis of AD” that seems more relevant to the development of the sporadic form of the disease than to the familial form (Krstic and Knuesel, 2013) (Figure 1).

Inflammation is a complex biological response of the immune system to harmful stimuli caused by chemical, physical, and biological factors. Although not only triggered by infection, inflammation secondary to infection plays a key role in the etiopathogenesis of AD progression (Ashraf et al., 2018). Infection-induced systemic inflammation is characterized by acute or chronic activation of a dysregulated host immune response, and the signals are not only restricted locally but also have potential systemic effects (Thorburn et al., 2018). C-reactive protein (CRP) is an important component of the innate immune system that is also used as a biomarker of inflammation (Kuo et al., 2005). The levels of this acute-phase reactant are elevated in bacterial and viral infections (Hu et al., 2017; Vasileva and Badawi, 2019). Many population-based prospective studies have suggested the association of CRP levels with the development of cognitive decline, especially AD (Duong et al., 1998; McGeer et al., 2000).

During the past decade, several studies have documented the possible contribution of peripheral infection and the role of peripheral immune activation in the progression of AD pathology (Kamer et al., 2008; Cao and Zheng, 2018; Choi et al., 2019). Infiltrating peripheral myeloid cells participate in Aβ clearance, as well as in replacing ablated microglia, to adopt a microglia-like phenotype in the brain with limited phagocytic capacity (Cao and Zheng, 2018). A recent study demonstrated that oral *Porphyromonas gingivalis* infection in a rodent model exacerbated the production Aβ_1−42_. The same pathogen was also identified in AD patients brain (Dominy et al., 2019). Thus, the prominent molecular and cellular changes in the periphery might have significant role in AD progression (Abbayya et al., 2015).

Nevertheless, the Aβ clearance after an infection remains a largely unexplored area. Knowing the fact that infection followed by systemic inflammation is sometimes accompanied by organ dysfunction, liver and kidney dysfunction need to be considered (Fujishima, 2016). However, the liver and kidney are the primary organs involved in the elimination of peripheral Aβ peptide. Thus, the major question remains: what is the fate of Aβ after infection? To answer this question, it would be necessary to gain a deeper insight into the post infection pathway of Aβ clearance.

Systemic inflammation induced by different infectious etiologies supports the amyloid hypothesis, inflammatory hypothesis, and antimicrobial hypothesis of AD. Thus, the accumulated knowledge, views and hypotheses from recent findings explains the infectious origin as one of the risk factors of AD progression. Although the molecular cascade that links systemic inflammation and neuroinflammation is still enigmatic, the possible modules that occur after infection, which lead to long-term impairment and brain dysfunction that ultimately trigger AD pathology, may include the following: Invading microorganisms escalate the peripheral Aβ load, a necessary step to neutralize and eliminate the pathogen from the peripheral environment. The peripherally produced Aβ and cytokines enter the CNS as systemic inflammation is able to increase BBB permeability. An increase in RAGE expression during systemic inflammation also facilitates the transport of Aβ to the central compartment. Finally, the entry of foreign substances triggers brain-immune system crosstalk, which in turn leads to activation of microglia/ astrocytes and local production of inflammatory mediators and reactive species (Figure 1). Further comprehension of these mechanisms with newer insights is warranted to develop a strategy for the potential advancement of therapeutics for infection-induced AD progression.

## Author Contributions

VG wrote the manuscript and proof the manuscript. FM, FP, and FD-P critically reviewed the manuscript. TB devised the main conceptual ideas and proof outline and designed the figure.

### Conflict of Interest Statement

The authors declare that the research was conducted in the absence of any commercial or financial relationships that could be construed as a potential conflict of interest.
